# Focused ultrasound activates voltage-gated calcium channels through depolarizing TRPC1 sodium currents in kidney and skeletal muscle

**DOI:** 10.7150/thno.33876

**Published:** 2019-07-28

**Authors:** Scott R. Burks, Rebecca M. Lorsung, Matthew E. Nagle, Tsang-Wei Tu, Joseph A. Frank

**Affiliations:** 1 Frank Laboratory, National Institutes of Health Clinical Center, Bethesda, MD 20892; 2 Department of Radiology, Howard University College of Medicine, Washington, DC 20059; 3 National Institute of Biomedical Imaging and Bioengineering, Bethesda, MD 20892

**Keywords:** focused ultrasound, Transient Receptor Potential Channel 1, Voltage-Gated Calcium Channels, Mechanotransduction, Calcium signaling, Depolarization, Acoustic radiation force, Cavitation

## Abstract

Pulsed focused ultrasound (pFUS) technology is being developed for clinical neuro/immune modulation and regenerative medicine. Biological signal transduction of pFUS forces can require mechanosensitive or voltage-gated plasma membrane ion channels. Previous studies suggested pFUS is capable of activating either channel type, but their mechanistic relationship remains ambiguous. We demonstrated pFUS bioeffects increased mesenchymal stem cell tropism (MSC) by altering molecular microenvironments through cyclooxygenase-2 (COX2)-dependent pathways. This study explored specific relationships between mechanosensitive and voltage-gated Ca^2+^ channels (VGCC) to initiate pFUS bioeffects that increase stem cell tropism.

**Methods:** Murine kidneys and hamstring were given pFUS (1.15 or 1.125 MHz; 4MPa peak rarefactional pressure) under ultrasound or magnetic resonance imaging guidance. Cavitation and tissue displacement were measure by hydrophone and ultrasound radiofrequency data, respectively. Elastic modeling was performed from displacement measurements. COX2 expression and MSC tropism were evaluated in the presence of pharmacological ion channel inhibitors or in transient-receptor-potential-channel-1 (TRPC1)-deficient mice. Immunohistochemistry and co-immunoprecipitation examined physical channel relationships. Fluorescent ionophore imaging of cultured C2C12 muscle cells or TCMK1 kidney cells probed physiological interactions.

**Results:** pFUS induced tissue deformations resulting in kPa-scale forces suggesting mechanical activation of pFUS-induced bioeffects. Inhibiting VGCC or TRPC1 *in vivo* blocked pFUS-induced COX2 upregulation and MSC tropism to kidneys and muscle. A TRPC1/VGCC complex was observed in plasma membranes. VGCC or TRPC1 suppression blocked pFUS-induced Ca^2+^ transients in TCMK1 and C2C12 cells. Additionally, Ca^2+^ transients were blocked by reducing transmembrane Na^+^ potentials and observed Na^+^ transients were diminished by genetic TRPC1 suppression.

**Conclusion:** This study suggests that pFUS acoustic radiation forces mechanically activate a Na^+^-containing TRPC1 current upstream of VGCC rather than directly opening VGCC. The electrogenic function of TRPC1 provides potential mechanistic insight into other pFUS techniques for physiological modulation and optimization strategies for clinical implementation.

## INTRODUCTION

High intensity focused ultrasound (HIFU) is a therapeutic modality that can be targeted precisely in deep body structures under image guidance with minimal effects on intervening tissues 1. Pulsed focused ultrasound (pFUS) is generally non-ablative and exerts mechanical forces of acoustic radiation forces (ARF) and cavitation on tissues 2, 3. Cavitation forces can be generated by pFUS interactions with dissolved tissue gases (e.g. during histotripsy) 4 or applied in conjunction with intravascular microbubble (MB) agents 5. Sonomechanical forces of ultrasound elicit a range of desirable and reproducible bioeffects on cells and tissues 6. Ultrasound bioeffects can be harnessed for a range of medical applications including blood-brain-barrier disruption for delivery of neurotherapeutics 7, neuromodulation 8, tumor immunomodulation 9, hemodynamic alterations 10, and regenerative medicine applications 11-17.

We previously demonstrated increased homing of intravenously-infused mesenchymal stromal cells (MSC) to sonicated tissues following pFUS to kidneys 11-14 or skeletal muscle 15-17. Using pFUS as a neoadjuvant prior to MSC infusions led to improved outcomes in diseased muscle and kidneys compared to MSC infusions alone 12, 17. Tropism of infused MSC occurred in response to pFUS upregulating expression of cytokines, chemokines, trophic factors (CCTF) and cell adhesion molecules (CAM) in treated tissues^18^. We demonstrated that CCTF and CAM upregulation by mechanical pFUS forces depended on nuclear factor κ-B (NFκB) and downstream cyclooxygenase-2 (COX2) upregulation 11, 16. NFκB signaling is canonically activated by elevated intracellular Ca^2+^ concentrations 19, 20, which would be generated by pFUS in this case.

Ultrasound mechanical forces have been shown to generate intracellular Ca^2+^ transients in several cell types 21. In addition to MSC homing, modulating intracellular Ca^2+^ dynamics is critical for pFUS applications such as neuromodulation in the brain 22 or immunotherapy by tumor sonication 23. However, mechanistic understandings of how pFUS forces initiate Ca^2+^ signaling at the plasma membrane remain relatively unknown. There are several reports of pFUS activating mechanically-sensitive cation channels directly through ARF or indirectly through MB cavitation forces. These demonstrations include modulating the transient receptor potential (TRP) 4 24 or the degenerin/epithelial Na^+^ channel (DEG/ENaC) MEC-4 25 in* Caenorhabditis elegans*, mechanically-sensitive human Na^+^ (Na_V_1.5) and K^+^ (K2P family) channels expressed in *xenopus* oocytes 26, and piezo1 channels in transfected human T-cells 27 or human embryonic kidney cells 28. Many of these studies employed highly-engineered experimental systems, but nonetheless, these channels are potentially involved in mechanically-sensitive ion fluxes that drive native biological responses to pFUS. Moreover, voltage-gated calcium channels (VGCC) and voltage-gated sodium channels (VGSC) in the plasma membrane have been shown to open following FUS 22, 29. Direct activation of voltage-gate ion channels by pFUS has been investigated, but primarily through theoretical modeling with limited physical evidence 30, 31. Modeling does suggest pFUS may directly activate voltage-gated channels (including VGCC 32) by altering the electrical properties of the plasma membrane. All of these previous studies have largely investigated isolated interactions between US and a particular channel type. They have not thoroughly explored the possibility of a mechanistic relationship between voltage-gated and mechanically-gated channels in the propagation of pFUS bioeffects.

This study investigated how voltage-gated and mechanically-gated plasma membrane ion channels interact to generate intracellular Ca^2+^ signaling following pFUS to mouse kidneys and skeletal muscle. We demonstrated that pFUS-based approaches can be utilized in these two tissues to improve cellular therapies and they also represent excitable and non-excitable cell types. We used pharmacological or genetic manipulations *in vivo* to delineate the necessity of the transient receptor potential channel 1 (TRPC1) and verapamil-sensitive VGCC to generate pFUS molecular responses that increase MSC tropism. To validate the *in vivo* results and explore the interdependencies between channel types, we identified a population of TRPC1 proteins that complexed with long-lasting (L)-type Ca^2+^ channels in the plasma membranes of kidney and muscle cells. *In vitro* ionophore imaging of kidney and muscle cells during pFUS revealed that this TRPC1 population was activated upstream of VGCC activation and TRPC1 currents depolarized the membrane to activate VGCC and generate cytosolic Ca^2+^ transients. This study presents a unifying mechanism to explain how both mechanically- and voltage-gated channels could be required to propagate Ca^2+^-dependent bioeffects of pFUS.

## RESULTS

### Physical effects of pFUS in muscle and kidney

**FIGURE 1:**
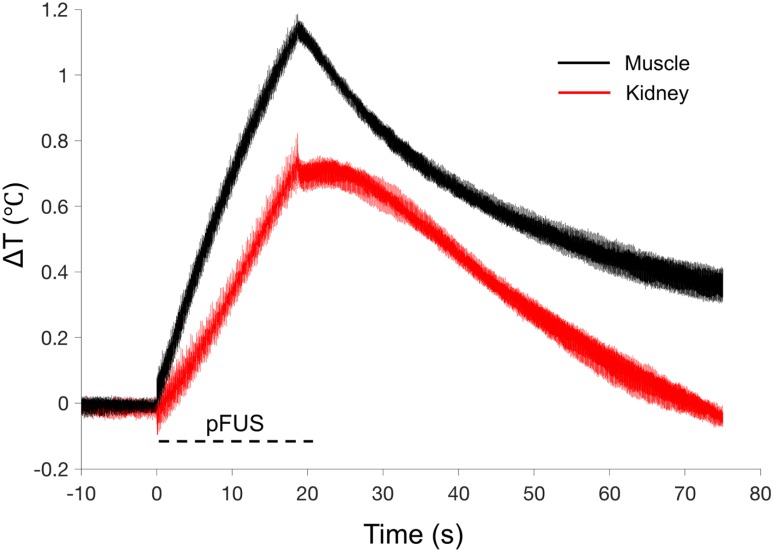
** Thermal effects of pFUS in murine kidney and hamstring muscle.** Average temperature changes during pFUS sonication of muscle (black line) and kidney (red line) during pFUS. Black dashed line indicates time during sonication (n=9 sonications per tissue type). The range of temperatures at the onset of each pFUS treatment was 36.106-37.224 ºC for kidney and 35.104-37.409 ºC for muscle.

**Figure 2:**
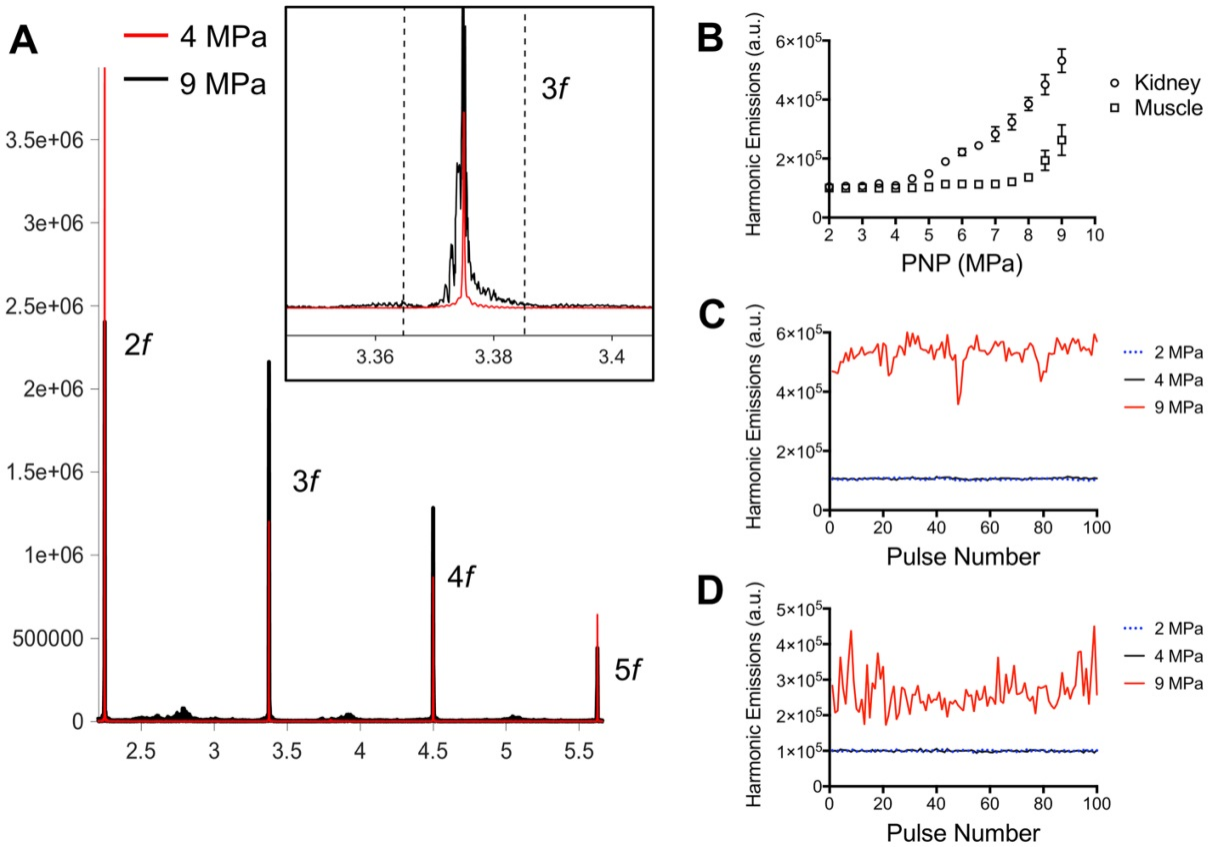
** Acoustic emissions during pFUS sonications of muscle and kidney. A)** Representative hydrophone spectra showing emissions at 2*f-*5*f* of the fundamental frequency (1.125 MHz) during pFUS to kidney tissue at 4 (red) or 9 (black) MPa PNP. Inset example show detail of the emissions at 3.375 MHz and black dashed lines indicated the bounds of the 20 kHz window where FFT amplitudes were integrated. The total FFT amplitude measured within 20 kHz windows around 2*f*, 3*f*, 4*f*, and 5*f* represented the value for “Harmonic Emissions” displayed in B-D. **B)** Harmonic emissions from 20 kHz windows around 2*f-*5*f* as a function of PNP in muscle or kidney. Previously unsonicated muscle or kidneys received 100 10 ms-pulses at each PNP. Emissions at 4 MPa in both tissues were statistically similar to emissions at 2 MPa. Increases in emission amplitudes were detected at PNP ≥5 MPa in kidneys and ≥ 8.5 MPa in muscle. Harmonic emissions in **C)** kidney or **D)** muscle as a function of pulse number during 100 pulse treatments at 2, 4, or 9 MPa PNP. Values for 4 MPa are similar to those at 2 MPa.

pFUS treatments (100 10-ms pulses) to kidney and muscle performed with a 1.15 MHz transducer and PNP of 4MPa with a duty cycle 5% resulted in peak temperature changes of ~1 ºC (1.1 ºC for muscle and 0.7 ºC for kidney; n=9 pFUS treatments per tissue type) (Figure 1). Acoustic spectra were acquired *in vivo* by hydrophone during sonications at 1.125 MHz and analyzed for acoustic emissions within 10 kHz of the frequencies corresponding to 2*f*-5*f* of the fundamental frequency (2.25, 3.375, 4.5, and 5.625 MHz, respectively) (Figure 2A). These acoustic emissions were measured across a range of PNP (2-9 MPa in 0.5 MPa increments). At each PNP, kidney or muscle received 100 10-ms pulses. The integrated amplitude values at 4 MPa in kidney and muscle were similar to those measured between <4 MPa (Figure 2B). However, increased acoustic emissions were detected at PNP ≥5 MPa in kidneys and ≥ 8.5 MPa in muscle. Plotting emission amplitudes as a function of pulse number during sonication revealed no differences between 2 or 4 MPa, but increased values are detected in all pulses at 9 MPa for both muscle and kidney (Figure 2C-D).

### Inhibiting mechanically- or voltage-gated Ca^2+^ channels or TRPC1 suppresses COX2 expression and MSC homing in pFUS-treated tissues

**FIGURE 4:**
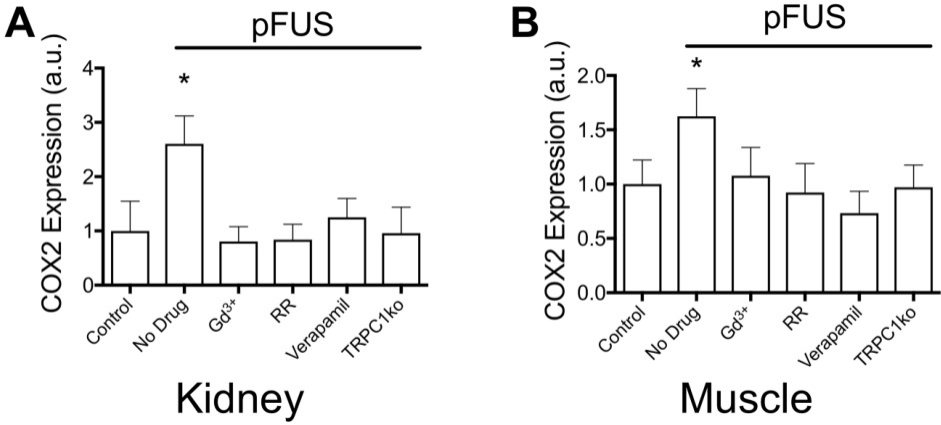
** COX2 expression in (A) kidney and (B) muscle homogenates at 6 hours post-pFUS.** COX2 expression increased when pFUS is administered to muscle or kidney of mice that receive no drugs. COX2 expression following pFUS was suppressed in wild-type mice that received intravenous GdCl_3_ (300 μg/kg), Ruthenium Red (RR; 22 mg/kg), or intraperitoneal verapamil (5 mg/kg) prior to pFUS and untreated TRPC1-KO mice (n=6 mice per group; separate cohorts were used for muscle and kidney treatments) (* p<0.05 by ANOVA). For presentation, control samples represent values from unsonicated tissues in mice that received no drugs or TRPC1-ko. These values were not significantly different from unsonicated tissues in mice that received drugs or TRPC1-ko (See Supplemental Data).

We previously characterized the mechanotransductive responses to pFUS in the context of NFκB and COX2 upregulation increasing CCTF and CAM on vascular endothelium that resulted in MSC homing to targeted tissues 16. COX2 expression was easily and reliably measurable and therefore, it was used as a proxy for the molecular CCTF response to pFUS. COX2 expression in kidney (Figure 4A) and muscle (Figure 4B) was measured at 6 hours post-pFUS in wild-type (WT) or TRPC1-ko mice that were treated with pFUS alone (4MPa) or in combination with different intravenously-administered agents prior to sonication (n = 6 mice/group; separate cohorts were used for kidney and muscle measurements). COX2 was significantly increased by pFUS 1.6 fold in muscle and 2.7 fold in kidney in control WT mice that received control saline infusions (p<0.05 by ANOVA). However, when mice were given IV GdCl_3_ (Gd^3+^ = 300 μg/kg) or Ruthenium Red (RR = 22 mg/kg) during pFUS, COX2 expression was not elevated at 6 hours post-sonication (p>0.05 by ANOVA). Gd^3+^ and RR indiscriminately interfere with mechanically-gated or voltage-gated cation channels 33-35. Intravenous verapamil (5 mg/kg) was given prior to pFUS to specifically inhibit VGCC in muscle and kidneys resulting in no increased COX2 expression in either tissue. Lacking a specific pharmacological inhibitor for mechanically-gated channels, we identified TRPC1 as a candidate mechanosensitive receptor (see Discussion). pFUS did not increase COX2 expression in muscle or kidney of TRPC1-ko mice (TRPC1 mice received no pharmacological manipulation). Moreover, baseline COX2 expression levels (no sonication) following pharmacological manipulation or TRPC1-ko alone were not significantly different than COX2 levels in control tissues (wild-type mice that did not receive drugs or sonication) (Supplemental Data). The TRPC1-ko mouse was engineered from the sv129 strain and to control for potential strain differences between the C3H and the sv129 background, pFUS was administered to muscle and kidney of sv129 mice. pFUS was found to upregulate COX2 in kidney and muscle to similar levels observed in the corresponding C3H mouse tissues (Figure S1).

**FIGURE 5:**
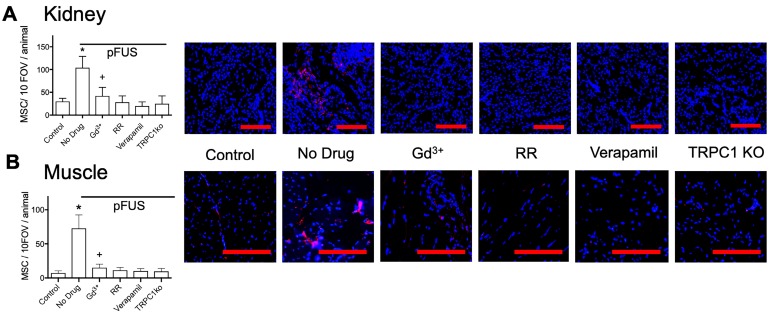
** MSC homing to (A) kidney and (B) muscle at 24 hours post-pFUS.** Mice received IV-infusions of 10^6^ human MSC 4 h post-pFUS and then were euthanized the following day. Kidney and muscle histology were stained for human mitochondria (red) to detect and quantify MSC (nuclei shown in blue). MSC were quantified in 10 fields-of-view in each of 3 histological slices per mouse from 3 mice. pFUS significantly increased MSC homing to wild-type kidneys and muscles, but homing was significantly suppressed in wild-type mice that received intravenous GdCl_3_ (300 μg/kg), Ruthenium Red (RR; 22 mg/kg), or intraperitoneal verapamil (5 mg/kg) prior to pFUS and untreated TRPC1-KO mice. Mice treated with GdCl_3_ had significantly more cells than control mice (no pFUS), but still had significantly fewer MSC than pFUS-treated mice that received no drug (*, ^+^ = p<0.05 by ANOVA) (scale bars represent 100 μm for kidney images and 50 μm for muscle images). For presentation, control samples represent values from unsonicated tissues in mice that received no drugs or TRPC1-ko. These values were not significantly different from unsonicated tissues in mice that received drugs or TRPC1-ko (See Supplemental Data).

Next, we investigated if these exogenous agents or if TRPC1 deficiency would also reduce tropism of MSC to pFUS-treated kidney or muscle. C3H mice (n=3 mice per group; separate cohorts were used for kidney and muscle) were administered the exogenous agents described above followed by pFUS to muscle or kidney. TRPC1-ko mice were given pFUS to the muscle or kidney without exogenous pharmacological agents to probe the involvement of TRPC1. In these experiments, IV infusions of 10^6^ human MSC were administered to all mice 4 hours after sonication (Figure 5). At 24-hour post-pFUS, muscle and kidneys were harvested from mice and immunostained for human mitochondria to detect MSC. pFUS-treated kidneys of C3H mice accumulated ~4 times more MSC as compared to untreated control kidneys, while pFUS-treated muscle in C3H mice accumulated ~7.5 times more MSC than untreated control muscle (p<0.05 by ANOVA). These observations are consistent with our previous studies of MSC homing to healthy tissues following pFUS 11, 13, 15, 16. Compared to control WT mice that received only pFUS (no agents), human MSC homing to pFUS-treated muscle or kidney was significantly inhibited by Gd^3+^, RR, verapamil, or TRPC1-ko (p<0.05 by ANOVA). The magnitudes of pFUS-induced MSC homing under each condition essentially mirrored the changes in COX2 expression. Of note, the number of cells in kidney and muscle in mice receiving Gd^3+^ was significantly greater than control levels, but also significantly less than mice receiving no agent (p<0.05 by ANOVA). Baseline quantities of MSC homing (no sonication) following pharmacological manipulation or TRPC1-ko were not significantly different than levels of MSC homing to control tissues (wild-type mice that did not receive drugs or sonication) (Figure S2). Increased homing was also observed following pFUS to kidneys and muscle in sv129 mice which are the background strain for the TRPC1-ko mouse (Figure S3).

### Intracellular Ca^2+^ signaling is necessary pFUS-stimulated COX2 expression in TCMK1 and C2C12 cells

**FIGURE 6:**
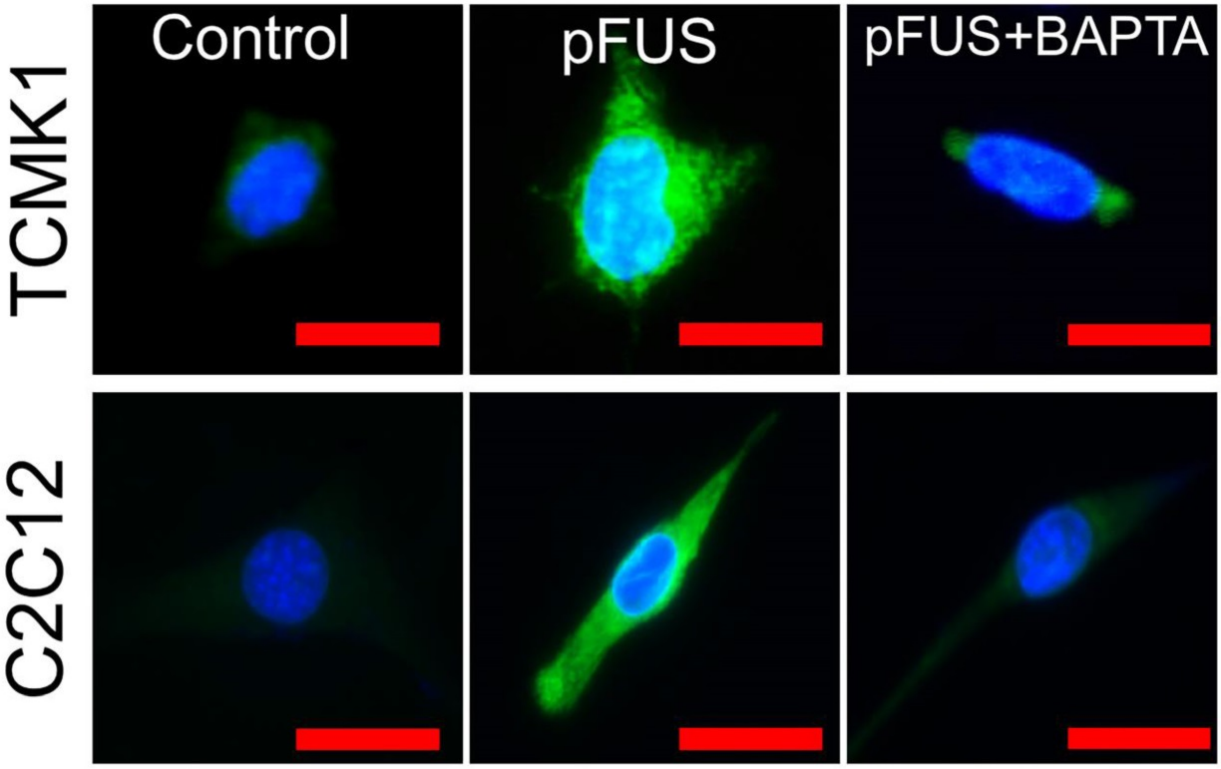
** COX2 upregulation by pFUS in TCMK1 and C2C12 cells is dependent on intracellular Ca^2+^.** Cultured TCMK1 or C2C12 cells were treated with pFUS and then immunostained for COX2 (green) 24 hours post-treatment (nuclei shown in blue). pFUS upregulated COX2 in both cell types and upregulations were blocked when both cell types were loaded with BAPTA-AM prior to pFUS (scale bars represent 10 μm).

To explicitly demonstrate the dependency of pFUS-induced COX2 expression on intracellular Ca^2+^, we sonicated cultured C2C12 cells (murine myoblasts) following differentiation into myotubes and TCMK1, a murine tubular epithelial cell line. pFUS-treated TCMK1 or C2C12 cells exhibited upregulated COX2 expression by immunostaining at 24 hours post-pFUS (Figure 6; experiments performed in triplicate). Other groups of these cells were sonicated after being incubated with 1,2-bis(*o*-aminophenoxy)ethane-*N,N,N',N'*-tetraacetic acid tetra(acetoxymethyl) ester (BAPTA-AM), a cell-permeable Ca^2+^-specific chelator. While BAPTA-AM did not further reduce baseline COX2 (Figure S4), it prevented pFUS from upregulate COX2 in either cell type.

### TRPC1 and L-type Ca^2+^ channels form complexes at the plasma membranes of skeletal muscle and kidney tubular cells

**FIGURE 7:**
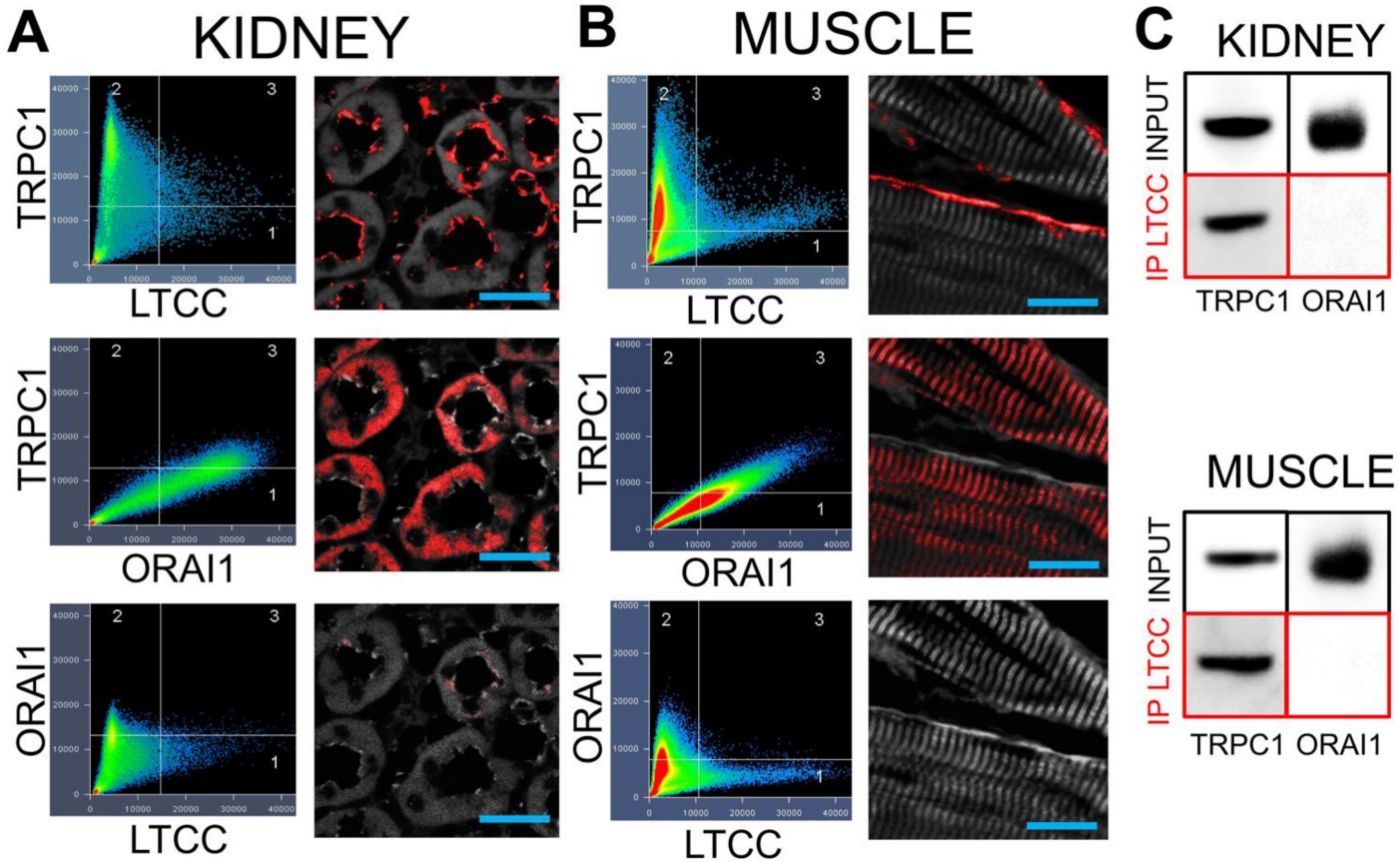
** TRPC1 and LTCC form complexes without ORAI1 in kidneys and muscle.** Confocal imaging for TRPC1, LTCC, and ORAI in **(A)** kidney and **(B)** muscle. Identical intensity thresholds were used for all groups within each tissue type to determine pixel colocalization (quadrant 3 in each image). Colocalized pixels for each comparison are shown in red and background pixels shown in grey. Comparing TRPC1 and LTCC reveals colocalizations on what appears to be the apical surfaces of tubular epithelium and the plasma membrane of skeletal muscle fibers. Substantial colocalization of TRPC1 and ORAI1 is observed, but in different cellular regions than the TRPC1/LTCC pixels. Lastly, there is essentially no colocalization between LTCC and ORAI1 (scale bars represent 20 μm). **C)** Immunoprecipitation of the LTCC from both tissue types precipitates associated TRPC1 molecules, but not ORAI1 molecules.

Demonstrating that VGCC, TRPC1, and intracellular Ca^2+^ are required for COX2 upregulation by pFUS, we examined potential interactions between TRPC1 and VGCC as they would relate to Ca^2+^ influx. We hypothesized that interacting channels would reside near each other in the cell and understanding structural relationships might give insight into function, specifically the sequence in which the channels opened following pFUS. Immunohistochemistry was performed on muscle and kidney from naïve C3H mice to investigate cellular localization of protein complexes. Confocal microscopy revealed a small population of TRPC1 proteins colocalizing with long-lasting (L)-type Ca^2+^ channels (LTCC) at the plasma membranes of renal tubular lumens (Figure 7A) and skeletal muscle fibers (Figure 7B).

TRPC1 is involved in multiple Ca^2+^ regulation processes. Notably, TRPC1 is a molecular component of store operated Ca^2+^ entry (SOCE) complexes 36 that refills depleted intracellular Ca^2+^ stores. Since SOCE would occur downstream of VGCC activation, we investigated whether the TRPC1 proteins in TRPC1/LTCC complexes had the requisite molecular components to act as SOCE channels. Among other components SOCE complexes contain TRPC1 and the Ca^2+^ release-activated Ca^2+^ channel protein 1 (ORAI1) 36 so immunostaining was performed for ORAI1 as well. Colocalization of TRPC1 and ORAI1 was observed, but in different parts of kidney or muscle cells as compared to TRPC1/LTCC complexes. ORAI1 did not colocalize with LTCC in either tissue type. Co-immunoprecipitation of LTCC from muscle and kidney homogenates further confirmed that LTCC are physically associated with TRPC1, but not ORAI1 (Figure 7C). This result indicates that the TRPC1 proteins that are complexed with LTCC are unlikely to function as SOCE channels since ORAI1 is not present in these complexes.

### VGCC currents in TCMK1 and C2C12 cells are activated by TRPC1-associated Na^+^ currents

**FIGURE 8:**
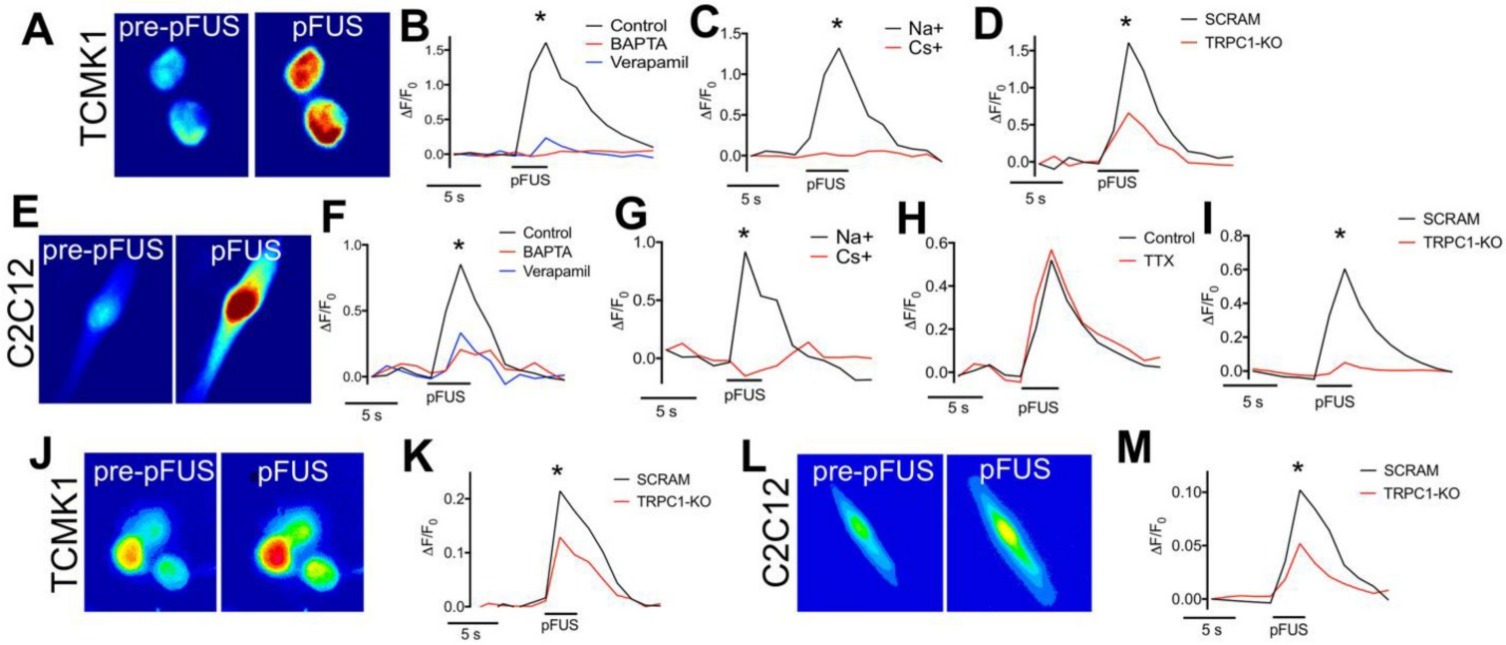
** Fluorescent ionophore imaging of TCMK1 and C2C12 cells during pFUS.** Representative Fluo-4 images of **(A)** TCMK1 and **(E)** C2C12 cells before and during pFUS. **(B, F)** Quantification of fluorescence intensities reveal that pFUS significantly increases Fluo-4 intensities in both cell types and Fluo-4 transients are effectively blocked loading with BAPTA-AM or incubating cells with 2 μM verapamil. **(C, G)** Partial replacement of Na^+^ in the extracellular solution with Cs^+^ also blocked Fluo-4 Ca^2+^ transients in both cell types, demonstrating dependence of Ca^2+^ transients on transmembrane Na^+^ potential. **(H)** Na^+^-dependent Fluo-4 Ca2^+^ transients were not affected by incubating C2C12 cells with 1 μM tetrodotoxin (TTX). **(D, I)** TRPC1 suppression by shRNA knockdown in each cell type resulted in diminished Fluo-4 Ca^2+^ transients compared to cells transfected with scramble control shRNA sequences. **(J, L)** Representative CoroNa transients during pFUS of TCMK1 and C2C12 cells. TRPC1 suppression in **(K)** TCMK1 cells or **(L)** C2C12 cells led to diminished CoroNa Green Na^+^ transients compared to cells transfected with scramble control shRNA sequences. (* = p<0.05 by t-test for pairwise comparisons or ANOVA for multiple comparisons).

To determine if Ca^2+^ transients occurred in TCMK1 and C2C12 cells during pFUS, the fluorescent Ca^2+^ ionophore Fluo-4-AM was added to culture media prior to pFUS (Figure 8A). Ca^2+^ transients were observed during sonication of TCMK1 (ΔF/F = ~1.5) and C2C12 (ΔF/F = ~0.5) cells (n = 30 cells/group; Figure 8B, F). Both cell types exhibited similar Ca^2+^ profiles. There were rapid increases in Fluo-4 fluorescence during pFUS that steadily decayed post-sonication. Incubating cells with BAPTA-AM prior to pFUS completely eliminated Fluo-4 transients (n = 30 cells/group) and sonicating either cell type in the presence of verapamil yielded a similarly undetectable Ca^2+^ transients (p>0.05 by ANOVA) (n = 30 cells/group) (Figure 8B, F).

Since TRPC1 conducts both Na^+^ and Ca^2+^, we investigated the potential role of Na^+^ currents by sonicating cells in a solution where 50% of the Na^+^ was replaced with Cs^+^, which reduced the permeability of Na^+^ through TRPC1 37. pFUS was unable to elicit Fluo-4 Ca^2+^ transients in either TCMK1 or C2C12 cells when Na^+^ potentials were reduced by Cs^+^ replacement, demonstrating that Ca^2+^ transients were depended on Na^+^ conductance (n = 30 cells/group) (Figure 8C, G). The potential involvement of voltage-gated Na^+^ channels was investigated in excitable skeletal muscle by sonicating C2C12 cells in the presence of tetrodotoxin (TTX; a voltage-gated Na^+^ channel inhibitor). TTX had no effect on Ca^2+^ transients during pFUS (n = 30 cells) (Figure 8I) suggesting that the Na^+^ dependence of Ca^2+^ transients did not involve voltage-gated Na^+^ channels. To evaluate the specific involvement of TRPC1 in pFUS responses, TCMK1 and C2C12 cells were transfected with vectors encoding shRNA against TRPC1 to knockdown protein expression (Figure S6). Compared to control cells that were transfected with scrambled shRNA sequences, TRPC1 knockdown significantly inhibited (p<0.05 by t-test) but did not eliminate Ca^2+^ transients measured by Fluo-4 in both TCMK1 and C2C12 cells (n = 30 cells/group). To investigate the dependence of Na^+^ conductance on TRPC1, the TRPC1-knockdown TCMK1 or C2C12 cells were loaded with the Na^+^ indicator CoroNa Green. TRPC1-knockdown cells (both TCMK1 and C2C12) exhibited significantly less intense fluorescence from Na^+^ indicators during pFUS than their respective control-transfected cells (p<0.05 by t-tests; Figure 8E, J).

## DISCUSSION

The study demonstrates that mechanically-gated TRPC1 and verapamil-sensitive VGCC are both required to transduce pFUS forces into intracellular Ca^2+^ signaling that upregulates COX2 and thus increases CCTF in the kidney and muscle microenvironments. Moreover, pFUS generates TRPC1 currents that secondarily activate complexed VGCC through local membrane depolarization. These results establish a basis for a unifying mechanism that reconciles previously disparate observations of how ultrasound activation of different plasma membrane channels.

To investigate how pFUS interacts within tissues, we evaluated the mechanical and thermal effects from ultrasound and found that bulk temperature increases were minimal during pFUS. While large temperature fluctuations were not observed during sonication pulses, we do not exclude the possibility of temperature spikes. Further studies will investigate potential temperature changes more precisely (e.g. utilizing thermocouples with faster response time, performing experiments outside of a water bath, etc.). Previous studies did not observe molecular signatures of heating beyond a physiological temperature range 16 or implicate thermal contributions to the molecular effects, so we hypothesized the molecular effects we observed were the result pFUS mechanical forces applied on the kidney and muscle. Mechanical pFUS effects occur through ARF or cavitation of bubbles. Bubbles can be either exogenous agents or at sufficiently large US pressures, form endogenously from dissolved tissue gases. Cavitation forces arise from stable oscillations of bubbles (non-inertial cavitation) or at elevated US pressures, unstable expansion/collapse of bubbles (inertial cavitation). Studies have utilized cavitation of exogenous MB agents to specifically activate mechanosensitive channels and generate intracellular Ca^2+^ fluxes. These demonstrations include peripheral neurons expressing the *c. elegans* homolog trp-4 and chimeric antigen receptor (CAR) T-cells expressing the human mechanosensitive channel piezo-1 24, 27. Our study did not use exogenous bubble agents and the measured acoustic emissions did not suggest bubble formation and cavitation in either tissue at 4 MPa, implying that the measured biological responses would be driven by ARF. Increased acoustic emissions in the kidney were observed at 4.5 MPa, but were not detected in muscle until PNP was >8 MPa. Several studies have characterized cavitation thresholds in biological tissues and shown a range of threshold pressures across different tissues 38-40. Since water content of kidney tissue is much greater than that of skeletal muscle, a lower cavitation threshold could be expected. Because increased acoustic emissions were observed in kidneys at PNP close to the 4 MPa employed in other experiments, studies with more sensitive hydrophones over the measured frequency range are still warranted to rule out that some renal cavitation was simply undetected at 4 MPa. It is also possible that other abdominal tissues in the pFUS beam path are the sources of acoustic emissions. For example, abdominal fat has been shown to have a relatively low cavitation threshold 38. The lack of a distinct acoustic cavitation signature however is suggestive that ARF are predominate during our sonication at 4 MPa and give rise to the observed bioeffects in kidney and muscle at these parameters. Other studies have shown that both the piezo-1 and MEC-4 channels can be activated using US parameters that emphasize ARF over cavitation 25, 28. While a specific mechanical activation threshold for TRPC1 could not be found, robust TRPC1 conductance has been reported at ~13 kPa of compression force 41, although this pressure was not determined to be a threshold value and it is possible that activation can be achieved at lower pressures. Negative tensile forces of 1 kPa have been shown to activate TRPC1 42 and even modest activation has been achieved at 0.6 kPa 43. Tissue during sonication would be subjected to multiple forces. Peak positive and negative pressure fluctuations in the pFUS field are in the MPa range and the FEA modeling suggests that forces (axial compression and lateral expansion) from tissue deformation during sonication can achieve >1 kPa which implicate TRPC1 activation could occur from tissue deformation as well. Future studies will evaluate molecular effects under a range of sonication parameters (different frequencies and pressures) to better understand the necessity or sufficiency of ARF and cavitation to induce desired bioeffects.

To investigate how pFUS forces are transduced by plasma membrane channels, experiments where Ca^2+^ channel signaling was genetically or pharmacologically altered in mice were performed. Under altered conditions, the ability for pFUS to upregulate COX2 expression and MSC homing to treated tissues was investigated. This study measured COX2 expression levels as a surrogate for the complex molecular changes that occur following pFUS, as we had previously observed that pharmacological interference with COX2 function or genetic knockout of COX2 protein prevented pFUS from enhancing MSC homing to treated tissues 11, 16. Initially, mice were given either GdCl_3_ or RR during pFUS to globally and non-specifically inhibit plasma membrane Ca^2+^ channels (i.e., voltage-gated and mechanosensitive channels)^44^, ^45^. Both agents inhibited COX2 upregulation and MSC homing compared to control mice that did not receive drugs. Of note, GdCl_3_ completely suppressed pFUS-induced upregulation of COX2 but only partially suppressed MSC homing to pFUS-treated tissues. It is possible that the Gd^3+^ dose was not sufficiently high to cause complete suppression. While COX2 expression levels are not increased by pFUS in the presence of this dose of Gd^3+^, incomplete suppression of Ca^2+^ fluxes could still lead to increased prostaglandin E_2_ without increasing COX2 expression. For example, Ca^2+^-dependent activation of phospholipase A_2_ can release a small quantity of membrane bound arachidonic acid^46^, which if metabolized by the physiological levels of COX2, might permit small increases in MSC homing. This observation highlights a potential pitfall of representing complex molecular changes following pFUS as simple changes in COX2 expression. While increasing COX2 expression following pFUS is necessary for robust MSC homing, future studies need to examine COX2 activity and production of prostaglandins rather than enzyme expression alone. However, these findings still highlight that some blockade of Ca^2+^ conductance across the plasma membrane using nonselective inhibition by Gd^3+^ or RR does block pFUS-induced elevations in COX2 and reduces MSC homing to sonicated tissues.

To probe potential involvement of more specific Ca^2+^ channel types, we selectively inhibited VGCC by verapamil 47 and observed similar inhibitory effects on COX2 expression and MSC homing. To probe mechanosensitive channels, the TRP channel family was identified as mechanosensitive candidates in mouse tissues. From our previous proteomic data 11, 16, we hypothesized that muscle and kidney could share similar mechanisms and expression profiling of TRP proteins in mouse tissues 48 and identified TRPC1 as being highly expressed in kidney and skeletal muscle. The lack of a specific pharmacological inhibitor for TRPC1 49 led us to investigate a TRPC1-ko mouse, where pFUS also failed to increase COX2 expression or induce homing of MSC.

Both TRPC1 and VGCC are required for cellular pFUS responses to upregulate COX2 and increase MSC homing and *in vitro* chelation of intracellular Ca^2+^ by BAPTA inhibited pFUS-induced COX2 upregulation. However, these observations create mechanistic ambiguity about the sequence of VGCC and TRPC1 activation to generate Ca^2+^ signaling within cells. Determining dependencies between TRPC1 and VGCC requires discussing how each functions with acknowledgement that cellular populations of TRPC1 are heterogenous and there are multiple ways TRPC1-containing complexes can modulate intracellular Ca^2+^ levels 50. VGCC (including the LTCC) activation is relatively straightforward—they open in response to membrane depolarization 51. Classically, they stimulate Ca^2+^-induced Ca^2+^ release (CICR) from the endoplasmic/sarcoplasmic reticulum (ER/SR) by activating nearby ryanodine receptors (RyR) in muscle cells 52. Moreover RyR are also expressed in kidney epithelium and modulate intracellular Ca^2+^ dynamics 53. TRPC1, however is a cation channel 54, 55 (or ion-channel-associated protein 56) that can be opened by mechanical forces 41 or intracellular Ca^2+^ store depletion 54, 55 (following CICR that would occur following VGCC activation). Thus, there are two possible relationships between TRPC1 and VGCC regarding cytosolic Ca^2+^ influx during pFUS: 1) Mechanical pFUS forces activate mechano-sensitive TRPC1 moieties first and their currents secondarily activate complexed VGCC by local membrane depolarization; or 2) VGCC are activated by pFUS through intramembrane cavitation which secondarily activate TRPC1-containing (by way of ER/SR store depletion that occurs following VGCC-activated CICR).

We hypothesized that relevant TRPC1 and VGCC interactions occurred between moieties that were in close physical proximity to each other. A small TRPC1 population was complexed with LTCC in the skeletal muscle plasma membranes and luminal plasma membranes of tubular epithelia. However, these complexes lacked the ORAI1 protein, which together with stromal interaction molecule 1 (STIM1), form the TRPC1-containing SOCE channel that opens to refill depleted intracellular Ca^2+^ stores 36. Numerous ORAI1/TRPC1 complexes were observed in parts of the cell away from the TRPC1/LTCC complexes. The lack of ORAI1 in the TRPC1/LTCC complexes suggests that this TRPC1 population is incapable of functioning as SOCE channels and therefore unlikely to be activated downstream of LTCC activation.

To further investigate the likelihood that pFUS activates this non-SOCE TRPC1 population prior to VGCC opening, ionophore imaging was performed on cultured TCMK1 kidney cells and differentiated C2C12 myotubes in the presence of BAPTA-AM (intracellular Ca^2+^ chelator) or verapamil (VGCC blocker). Verapamil essentially completely inhibited Ca^2+^ transients during pFUS. A near complete suppression of Ca^2+^ transients by verapamil block of VGCC was interesting and suggested that if TRPC1 proteins were activated upstream of VGCC, they would conduct primarily Na^+^. Indeed, a previous report of TRPC heterodimers reported TRPC1-containing heterodimers had increased Na^+^ permeability and reduced Ca^2+^ permeability 57. Should TRPC1 currents simply serve to depolarize VGCC during sonication, Na^+^ conductance would suffice. The involvement of Na^+^ conductance in pFUS-induced Ca^2+^ transients was examined by reducing transmembrane Na^+^ potentials by replacement in the extracellular solution with less-permeable Cs^+^. When Na^+^ potentials were reduced, pFUS generated less robust Ca^2+^ transients upon Fluo-4 imaging in both cell types, which indicated that Ca^2+^ transients depended upon Na^+^ currents. pFUS performed on C2C12 in the presence of TTX eliminated the potential for VGSC involvement in excitable muscle cells. Lastly, we silenced TRPC1 expression with shRNA in both cell types and observed diminished Ca^2+^ transients upon Fluo-4 imaging and diminished Na^+^ transients upon CoroNa Green imaging. Na^+^ ionophore imaging is especially informative and reveals specific TRPC1-associated Na^+^ currents occur during pFUS and are critical for membrane depolarization to activate VGCC. The concept that mechanosensitive channels can have electrogenic functions (i.e. voltage-gated channels are regulated by mechanically sensitive currents) an emerging mechanobiology hypothesis that is becoming supported by experimental observations 58.

**Figure 9:**
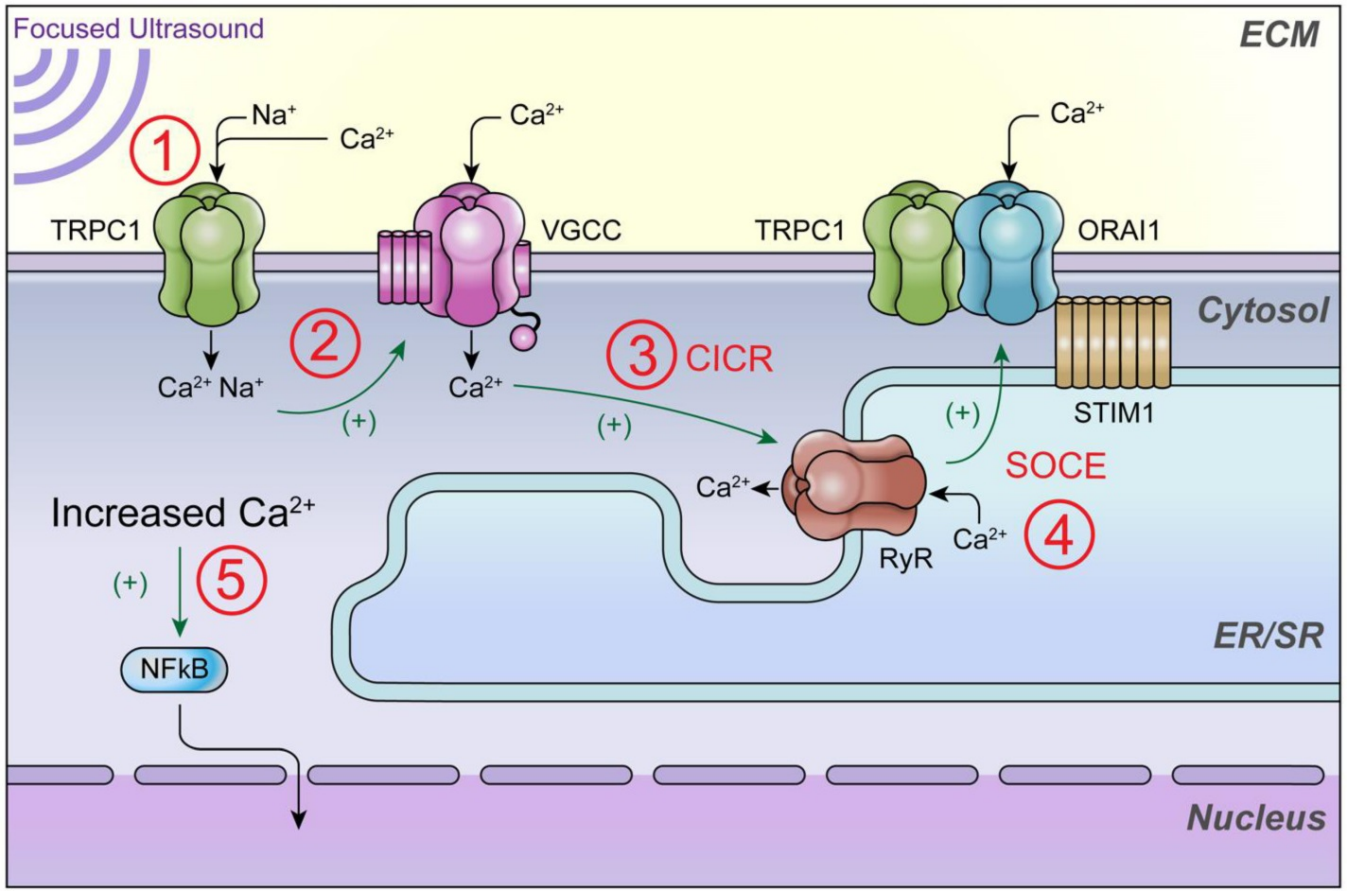
** Schematic of intracellular Ca^2+^ signaling that generates ultrasound bioeffects for enhanced MSC homing.** This study demonstrates that 1) pFUS mechanically activates cationic TRPC1 channels that conduct depolarizing currents and 2) activate nearby VCGG to amplify cytosolic Ca^2+^ concentrations. Other potential but uninvestigated downstream mediators of cytosolic Ca^2+^ influx could be 3) Ca^2+^-induced Ca^2+^-release (CICR) through ryanodine receptors (RyR) following VGCC activation and 4) the TRPC1/ORAI1/STIM1 complex, which may be involved in downstream store operated Ca^2+^ entry (SOCE) following potential ER/SR depletion. 5) The ultimate effect of increased cytosolic Ca^2+^ is activation of nuclear factor κ B (NFκB) which generates molecular responses (including COX2) necessary to generate the variety of cytokines, chemokines, trophic factors, and cell adhesion molecules that are necessary to induce tropism of infused MSC 16.

This study elucidates the order in which plasma membrane channels are activated during pFUS and provides insight into the biophysical mechanisms of how pFUS is sensed by cells. A non-SOCE TRPC1 complex that conducts Na^+^ to activate VGCC and initiate intracellular Ca^2+^ signaling (see illustrative schematic in Figure 9). The results of this study may help resolve disjointed observations regarding mechanotransduction of pFUS forces. Future studies are still need to further probe additional Ca^2+^ signaling events that could occur following VGCC activation and contribute to the total cytosolic Ca^2+^ transient that is observed. For example, it is unlikely that VGCC currents account for the entirety of the imageable Ca^2+^ transient. Other likely contributions to Fluo-4 fluorescence increases include VGCC initiating RyR-mediated CICR which could also be followed by SOCE through ORAI1/STIM1/TRPC1 complexes. This study only examined SOCE (ORAI1-containing) TRPC1 complexes to distinguish the non-SOCE TRPC1 population that conducts Na^+^ that is critical for VGCC activation. However, other populations of TRPC1 that comprise SOCE complexes could still operate well downstream of mechanical pFUS forces and therefore still play important roles in Ca^2+^-dependent NFκB/COX2 activation. Further studies will explore other possible molecular mediators of cytosolic Ca^2+^ flux to understand the remaining contributors to overall Ca^2+^ influx.

These findings may also shed light on other pFUS applications such as neuromodulation, where mechanistic understanding remains both incomplete and controversial. Evidence that pFUS alters membrane dynamics (i.e., intramembrane cavitation) to electrically activate VGCC, which has been predicted was not appreciated. Furthermore, modeling suggests that slight temperature increases also effect channel activation thresholds. We cannot entirely rule out thermal effects from pFUS, however the overall biological responses appear consistent between animal experiments conducted at 37 ºC or *in vitro* experiments conducted at room temperature. This observation suggests that temperature-independent pFUS activation occurs in this temperature range, but further investigation would be needed to accurately determine the role of temperature elevations at the plasma membrane. In excitable skeletal muscle cells, which would be more similar to neurons, we did not observe any direct activation of voltage-gated channels by pFUS. However, the diversity of both TRP and voltage-gated channels is entirely different in neurons and extending these observations to the neuromodulation studies in the brain will depend on the role of TRPC1 or other mechanosensitive channels such as TRPC4 or TRPC6, not to mention human tissues have additional neuronal mechanostretch receptors such as Piezo channels which could alter neuronal physiology during sonication. Immunotherapy augmentations by pFUS in the treatment of cancer and autoimmune diseases have also garnered recent attention 59, 60. It would also be important to elucidate the molecular mechanisms that could potentiate or enhance beneficial outcomes. pFUS could be an attractive neoadjuvant approach to stimulate immune responses to tumors as an alternative to other techniques where mechanical destructive (i.e., histotripsy) and/or radiofrequency or high intensity focused ultrasound thermal ablation of tissue would be undesirable (e.g. microsatellite or residual disease residing within “healthy” appearing tissues following other interventions or treatments). Image guided pFUS can be targeted alone or in conjunction with microbubble agents, as a non-destructive mechanical force to induce pro-inflammatory responses that stimulate both innate and adaptive immune cell tropism to sonicated tissues. Low intensity pFUS likely generates sterile inflammation responses to release of damage associated molecular patterns (DAMP) released from cells and successful modulation of the immune system would require manipulating cellular Ca^2+^ dynamics to activate NFκB pathways and stimulate pro-immune signal transduction 11, 16, 61.

In conclusion, pFUS is a technology with broadening appeal for numerous applications, but mechanistic understanding of bioeffects and how they arise from ultrasound/tissue interactions has lagged far behind biomedical implementation. This study further outlines the relationships between different classes of membrane channels and how they interact during pFUS to initiate intracellular Ca^2+^ signaling that that is a requisite for improving cellular therapies. The relationship we identified between channels with different gating properties could also provide insight into mechanisms that might be commonly related across different pFUS applications that aim to modulate physiology.

## METHODS

### Animals

Animal studies were approved by the Animal Care and Use Committee at the National Institutes of Health Clinical Center. All procedures were performed in accordance with relevant guidelines and regulations. Female C3H, SV129, or *Trpc1^tm1Lbi^*/Mmjax (TRPC1-knockout) mice were used in this study. All mice were from Charles River Laboratories (Wilmington, MA) or the Jackson Laboratory (Bar Harbor, ME). Mice were given free access to food and water.

### Pulsed Focused Ultrasound

pFUS was administered to murine kidneys or hamstrings under ultrasound imaging guidance by a VIFU 2000 and e-Cube 12 (Alpinion, Bothell, WA) or under magnetic resonance imaging guidance (3T Acheiva, Philips Healthcare) using an LP-100 (FUS Instruments, Toronto, Ontario, Canada). Mice were submerged in degassed water at 37 ºC with their dorsal aspect facing the transducer and AptFlex F28 acoustic absorbing material (Precision Acoustics, Dorset, UK) was placed in far field (i.e. on the ventral side of the mouse) to prevent acoustic reflections. pFUS was delivered using the following parameters: 1.15 MHz operating frequency, 4 MPa peak negative pressure, 10 ms pulse length, 5 Hz pulse repetition frequency, and 100 pulses per site. Sonication points were spaced 2 mm apart and covered the entire kidney or hamstring. Mice were given unilateral pFUS and the contralateral tissue was used as the untreated internal control. Mice receiving pFUS to kidneys did not receive pFUS to hamstrings and vice versa. Control mice were treated with sham pFUS exposures (transducer power = 0 W). Temperature measurements were made on the by inserting a thermocouple (Type K, 76 μm diameter, 1 m length) (RS Components, Corby, UK) into the sonicated tissue and placing it the center of the focal zone. Data were acquired with a digital multimeter (34465A; Keysight Technologies, Santa Rosa, CA) sampling at a rate of 100 Hz. For hydrophone measurements, a 1.125 MHz transducer with integrated hydrophone (lead-zirconate-titanate ceramic with a center frequency 0.5 MHz [FUS Instruments]) was used. Hydrophone signals were sampled by an oscilloscope at 20 MHz for the duration of each 10-ms pulse. Kidneys and hamstring muscles were targeted using T2-weighted imaging sequences described previously 61, 62.

*In vitro* pFUS was administered using a focused ultrasound transducer (H101, Sonic Concepts, Bothell WA) operating at 1.12 MHz using a function generator and amplifier that were controlled by LabView (National Instruments, Austin TX). The transducer was immersed in a manufacturer-supplied coupling cone containing degassed water and sealed with an ultrasound-transparent membrane. Transducer output was calibrated by hydrophone (HGL400; ONDA, Sunnyvale, CA). The coupling cone was place directly over the objective of an inverted epifluorescence microscope for treatment. The focal diameter of the transducer was 1.37 mm so the entire microscopic field of view was assumed to have uniform US distribution.

### Acoustic Data Acquisition and Processing

Hydrophone signals were processed offline in MATLAB (Mathworks, Natick, MA). Electrical signals were transformed using Fast Fourier Transforms (FFT). To quantify acoustic emissions, FFT amplitude values within 20 kHz windows around harmonics (i.e. harmonic frequency ± 10 kHz) were integrated. Integrated values from 20 kHz windows around emissions at 2.25, 3.375, 4.5, and 5.625 MHz (corresponding to 2f, 3f, 4f, and 5f, respectively) were totaled and plotted as function of ultrasound peak negative pressure.

RF data sampled every 10 μs were from imaging transducer elements in the center of the pFUS beam path. 1D cross-correlation analyses 2 at the near- and far-field surfaces of kidneys and hamstrings were performed to determine tissue compression and correct for structural displacement during sonication. Tissue compression data was used in simple elastic finite element analysis modeling performed as previously described 63 using the elastic moduli of muscle 64 and kidney 65 to determine axial and lateral strain amplitudes due to tissue deformation.

### Animal Drug Treatments

Some groups of mice received drugs in conjunction with pFUS exposures. GdCl_3_ (20 μmol/kg; Sigma-Aldrich, St. Louis, MO) 66 was slowly infused intravenously during the sonication. Ruthenium Red (12 μmol/kg; Sigma-Aldrich) 67 and Verapamil (5 mg/kg; Hospira, Lake Forest, IL) 68 were infused intraperitoneally 10-15 min prior to sonication.

### Cell Cultures and MSC Infusion

Human MSC were donated to the NIH Center for Bone Marrow Stromal Cell Transplantation under the clinical trial NCT01071577 (www.clinicaltrials.gov), which was approved by the NIH Clinical Center IRB and included informed consent from donors. MSC were cultured in α-minimum essential medium supplemented with 20% fetal bovine serum (FBS). MSC for this study were previously characterized for cell surface marker expression 12 and all experiments employed MSC at passage number 5 or less. MSC were cultured in 175 cm^2^ flasks and were allowed to reach ~80% confluence before use. For infusions into mice, MSC were resuspended to 10^7^ cells/mL in Hank's balanced salt solution (HBSS) without divalent ions that contained 10 U/mL sodium heparin. Approximately 4 h post-pFUS, mice were given an intravenous (iv) injection of sodium nitroprusside into the lateral tail vein (1 mg/kg in 0.9% saline)^69^. Immediately after sodium nitroprusside, 10^6^ MSC (in 100 μL) were infused into the opposite lateral tail vein.

For *in vitro* studies, murine TCMK1 kidney epithelium was cultured in Roswell Park Memorial Institute (RPMI) 1640 supplemented with 10% FBS and 1% penicillin/streptomycin. Murine C2C12 myoblasts were cultured in Dulbecco's minimum essential medium (DMEM) supplemented with 20% FBS and 1% penicillin/streptomycin. Prior to experimentation, C2C12 myoblasts were differentiated in DMEM containing 10% horse serum for 2 days. All cell culture reagents were from Life Technologies (Carlsbad, CA). TCMK1 and C2C12 cells were transfected with lentiviral particles containing plasmids encoding shRNA against murine TRPC1 or scrambled control sequences (Santa Cruz Biotechnology, Santa Cruz, CA) according to manufacturer protocols. Control and TRPC1 knockdown cells were cultured in the presence of 7.5 μg/mL puromycin (Santa Cruz). To chelate intracellular Ca^2+^ in some experiments, TCMK1 or C2C12 cells were loaded with 1,2-bis(*o*-aminophenoxy) ethane-*N,N,N',N'*-tetraacetic acid-acetoxymethyl ester (BAPTA-AM; Life Technologies) according to manufacturer protocols.

### Tissue Harvesting

For COX2 quantitation, sonicated muscles or kidneys were harvested from mice 4 h post-sonication. Tissues were homogenized in Tris-buffered saline (TBS) containing 0.05% Tween-20 and protease inhibitors (Roche, Basel, Switzerland). Hamstrings and kidneys were harvested from other mice that did not receive pFUS or MSC and were homogenized in the same buffer for co-immunoprecipitation analyses. For colocalization studies, normal untreated mice were perfused and muscle and kidney tissue were embedded in paraffin for immunohistochemistry. To quantify MSC, mice receiving kidney or muscle pFUS followed by MSC infusions were euthanized 24 h post-infusion. Mice were perfused and tissue was embedded in paraffin for MSC detection.

### Immunohistochemistry, MSC quantification, and Immunocytochemistry

See Table S1 for comprehensive listing of antibodies used, sources, and dilutions for each experiment. Tissue sections (10 μm) were deparaffinized in xylenes and rehydrated in graded ethanol. For MSC detection, slides were permeabilized with 0.1% Triton X-100. All slides were blocked with Superblock (Thermo) and incubated with primary antibodies overnight at 4 ºC. Fluorophore-conjugated secondary antibodies were incubated for 1 h at room temperature. Slides were coverslipped using ProLong Diamond antifade reagent containing 4', 6-diamidino-2-phenylindole (DAPI). Sections for colocalization studies were imaged by confocal microscopy with filter settings determined by the operating software (LSM 710, Zeiss, Oberkochen, Germany). Sections for MSC quantification were imaged using an Aperio ScanScope-FL (Leica Biosystems, Wetzlar, Germany). MSC were quantified as described previously under blinded conditions^11^-^17^.

For immunocytochemical detection of COX2 in TCMK1 and C2C12 cells, cells were fixed with 10% formalin for 15 minutes at room temperature, permeabilized with 0.1% Triton X-100, and blocked with SuperBlock. COX2 primary antibody was incubated for 3 h at room temperature and the secondary antibody was incubated for 30 min at room temperature. Cells were imaged using an inverted epifluorescence microscope (IXplore, Olympus, Tokyo, Japan).

### Protein Analyses

COX2 expression was determined in muscle and kidney homogenates by enzyme-linked immunosorbent assay (ELISA)(R&D Systems, Minneapolis, MN). Samples were plated at a concentration of 2 mg/mL and ELISA plates were developed according to manufacturer protocols.

Co-immunoprecipitation was performed on homogenates from normal and untreated muscle or kidneys were incubated for 1 h at room temperature with antibodies against L-type calcium channel subunits. Precipitation was performed using a Dynabeads Protein G immunoprecipitation Kit (Thermo). Precipitates were analyzed for the presence of TRPC1 and ORAI1 by Western Blotting.

Western blotting was performed to analyze co-immunoprecipiations and efficacy of TRPC1 knockdown. Samples under reducing conditions were electrophoresed on 4-12% polyacrylamide gels and transferred to nitrocellulose membranes. Blocking was done with SuperBlock and primary antibodies were incubated for 3 h at room temperature. Fluorophore-conjugated secondary antibodies were incubated for 1 h at room temperature. Blots were imaged on gel imager (ChemiDoc; BioRad, Hercules, CA).

### Fluorescent Ionophore Imaging

TCMK1 and C2C12 cells were loaded with Fluo4-AM or CoroNa Green-AM (Life Technologies) prior to imaging according to manufacturer protocols. Imaging was performed on an inverted epifluorescence microscope (IXplore, Olympus, Tokyo, Japan) in serum-free RPMI without phenol red (Thermo) during sonication at room temperature. For measurements utilizing exogenous agents to manipulate physiology, BAPTA-AM was loaded as described above immediately prior to loading with fluorescent ionophores. Other exogenous agents were added to the extracellular medium immediately before treatment. Verapamil (Sigma-Aldrich) was used at 2 μM and tetrodotoxin (TTX; Abcam, Cambridge, UK) was used at 2 pM. Altered Na^+^ potentials were achieved by diluting RPMI by 50% and then re-supplementing NaCl or an identical concentration of CsCl. Ca^2+^, Mg^2+^, K^+^, and Cl^-^ were also restored to levels described in the product formulation.

### Data Analyses and Presentation

Statistical analyses and data presentation were performed in Prism (GraphPad, La Jolla, CA). All data are presented as mean values ± SD. All statistical tests were 2-sided and p values <0.05 were considered significant. Pairwise comparisons were made using t-tests and multiple comparisons were made using one-way analysis of variance (ANOVA) with Bonferroni post-hoc analyses.

## Supplementary Material

Supplementary figures and tables.

## Figures and Tables

**FIGURE 3:**
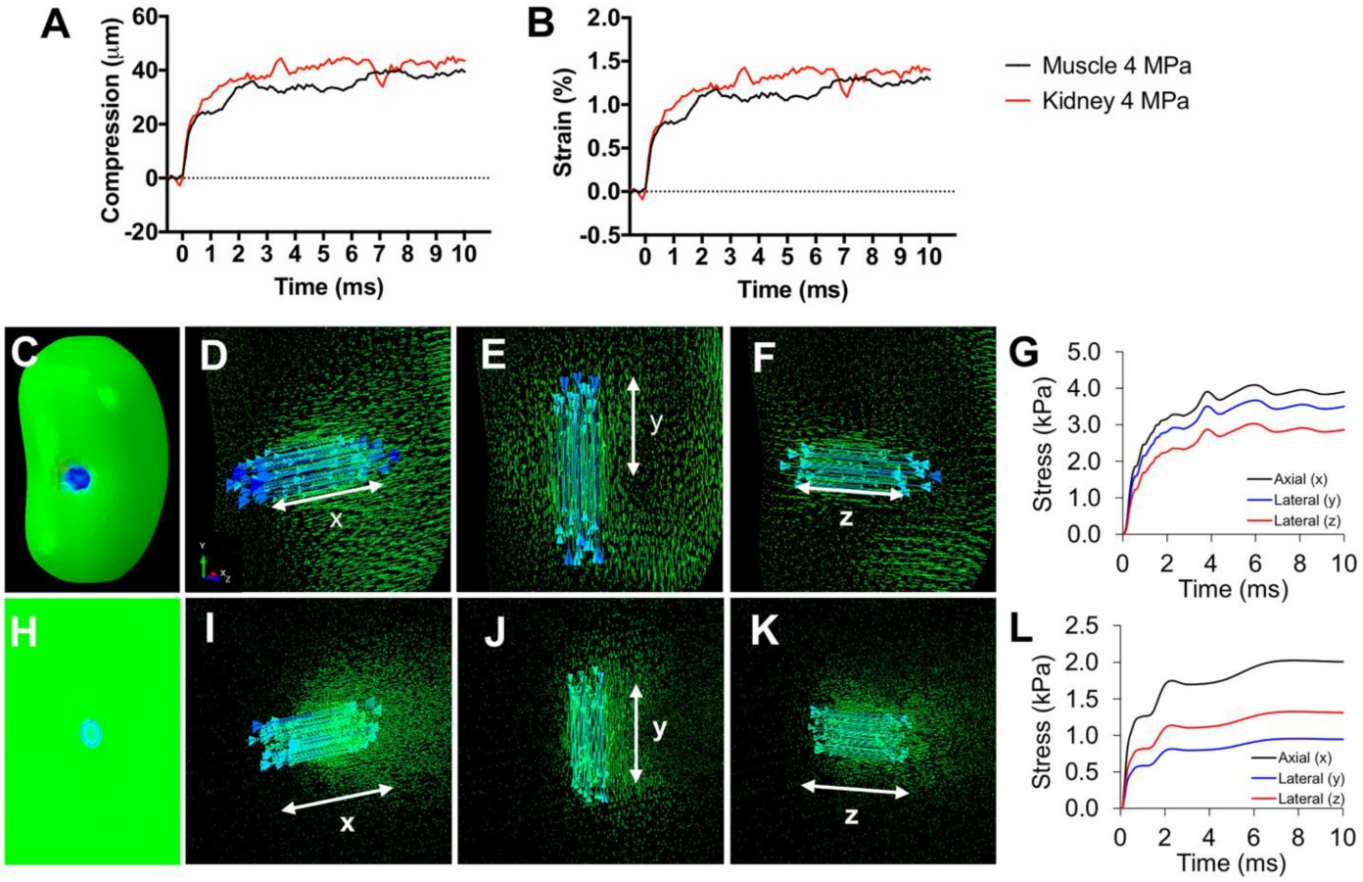
** Tissue strain and FEA modeling of tissue stresses during pFUS.** Representative traces of **A)** compression and **B)** axial strain of kidneys and muscle during 10 ms sonication.** C)** Contour map of a kidney showing axial principle stress component (axis of pFUS propagation). Vector maps of stresses are shown for the **(D)** axial and **(E, F)** both lateral directions indicated by white arrows. **G)** Principle stresses at the center of the pFUS focus during a 10 ms sonication of the kidney.** H)** Contour map of hamstring muscle showing axial principle stress component. Vector maps of stresses are shown for the **(I)** axial and **(J,K)** both lateral directions indicated by white arrows. **L)** Principle stresses at the center of the pFUS focus during a 10 ms sonication of the hamstring.
