# Focused ultrasound activates voltage-gated calcium channels through depolarizing TRPC1 sodium currents in kidney and skeletal muscle

**DOI:** 10.7150/thno.69908

**Published:** 2022-01-01

**Authors:** Scott R. Burks, Rebecca M. Lorsung, Matthew E. Nagle, Tsang-Wei Tu, Joseph A. Frank

**Affiliations:** 1 Frank Laboratory, National Institutes of Health Clinical Center, Bethesda, MD 20892; 2 Department of Radiology, Howard University College of Medicine, Washington, DC 20059; 3 National Institute of Biomedical Imaging and Bioengineering, Bethesda, MD 20892

The final production of this manuscript omitted text in the Results section to describe the findings presented in Figure 3. The following text was included in all previously-submitted versions of the manuscript and was peer-reviewed at the same time as the rest of manuscript. This text in quotations originally appeared under the subheading “Physical effects of pFUS in muscle and kidney”.

“Cross-correlation analyses of US radiofrequency data at 4 MPa PNP revealed that the average compression of kidney tissue (n=3) was ~45 μm by the end of the 10 ms pulse, which corresponded to a ~1.28% axial strain. Hamstring muscle compression (n=3) was ~40 μm at the end of the 10 ms pulse, which corresponded to a ~1.31% axial strain (Figure 3A-B). From the strain data and known elastic properties of each tissue, finite element analysis (FEA) modeling was conducted to estimate tissue stress in the axial dimension (along the axis of pFUS propagation) and resulting lateral stress in the orthogonal axes that result from tissue compression (Figure 3C-L). During a 10 ms pulse to the kidney a maximal axial stress of 3.9 kPa was estimated while maximal stresses in the lateral dimensions were 3.5 and 2.8 kPa. Similar modeling of the hamstring revealed a maximal axial stress of 2.0 kPa and maximal stresses in the lateral dimensions of 0.95 and 1.31 kPa.”

The authors apologize for this oversight in final review of the manuscript and this erratum does not in any way alter the findings or conclusions of the manuscript.

