# Supplementary material for: Focused ultrasound activates voltage-gated calcium channels through depolarizing TRPC1 sodium currents in kidney and skeletal muscle
Source: Theranostics. 2019 Jul 28;9(19):5517–31. doi: 10.7150/thno.33876 (PMC6735402; doi:10.7150/thno.33876)
Supplement: Supplementary file 1 — Supplementary figures and tables. [file thnov09p5517s1.pdf]

Supplemental Information for:

Focused ultrasound activates voltage-gated calcium channels through depolarizing TRPC1 sodium currents in kidney and skeletal muscle

Scott R. Burks[1], Rebecca M. Lorsung[1], Matthew E. Nagle[1], Tsang-Wei Tu[2], and Joseph A. Frank[1,3]

[1]Frank Laboratory, National Institutes of Health Clinical Center, Bethesda, MD 20892

[2]Department of Radiology, Howard University College of Medicine, Washington, DC 20059

[3]National Institute of Biomedical Imaging and Bioengineering, Bethesda, MD 20892

Running title: Ultrasound-Activated TRPC1 Currents Open Voltage-Gated Calcium Channels

Key Words: focused ultrasound, Transient Receptor Potential Channel 1, Voltage-Gated Calcium Channels, Mechanotransduction, Calcium signaling, Depolarization, Acoustic radiation force, Cavitation

Antibody	Vendor	Catalog Number	Application/Dilution
Human Mitochondria	Abcam	ab92824	IHC/1:200
TRPC1	Santa Cruz Biotechnology	sc-514685	WB/1:1000
			IHC/1:100
ORAI1	Abcam	ab86748	WB/1:500
	Sigma	SAB2500717	IHC/1:100
L-type Calcium channel α1D (kidney)	Santa Cruz Biotechnology	sc-515643	IP/1:20
			IHC/1:100
L-type Calcium channel α1S (muscle)	Santa Cruz Biotechnology	sc-514685	IP/1:20
			IHC/1:100
COX2	Abcam	ab15191	ICC/1:500
COX2 ELISA	R&D Systems	DYC4198	N/A

Supplemental Table 1. Antibody products, vendor information, and dilutions. Abbreviations: IHC, immunohistochemistry; WB, Western Blot; IP, immunoprecipitation; ICC, immunocytochemistry

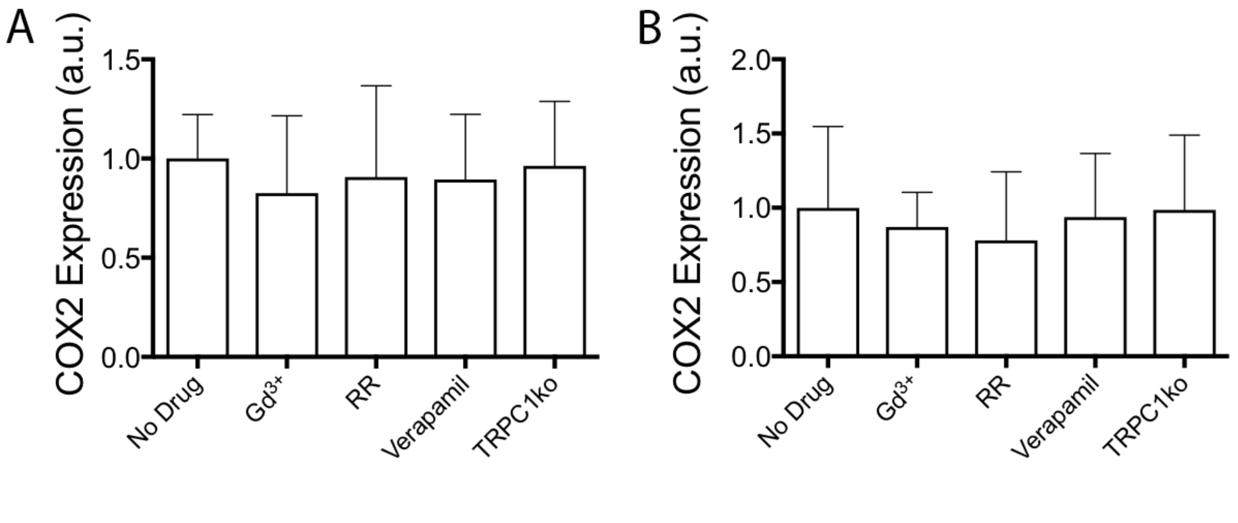

Supplemental Figure 1. Pharmacological or genetic manipulations in mice do not affect COX2 expression levels in unsonicated A) muscle or B) kidneys. For pharmacological manipulations, drugs were administered 10-15 minutes prior to a sham pFUS treatment (transducer power = 0 W). Tissues were harvested 4 hours post-treatment and COX2 expression was determined by ELISA. P values for all comparisons were >0.05 by ANOVA using Bonferroni post-hoc analyses (n=6 mice per group).

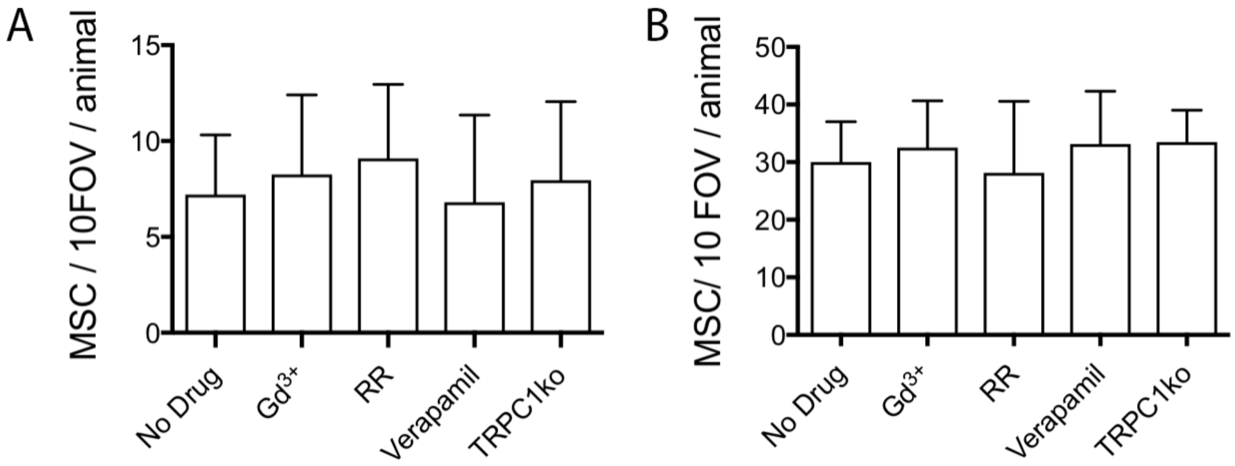

Supplemental Figure 2. Pharmacological or genetic manipulations in mice do not affect quantities of MSC homing to unsonicated A) muscle or B) kidneys. For pharmacological manipulations, drugs were administered 10-15 minutes prior to a sham pFUS treatment (transducer power = 0 W). MSC were infused into mice 4 hours after sham treatment and MSC were quantified in histological sections of tissue harvested 24 hours post-treatment. P values for all comparisons were >0.05 by ANOVA using Bonferroni post-hoc analyses (n=3 mice per group).

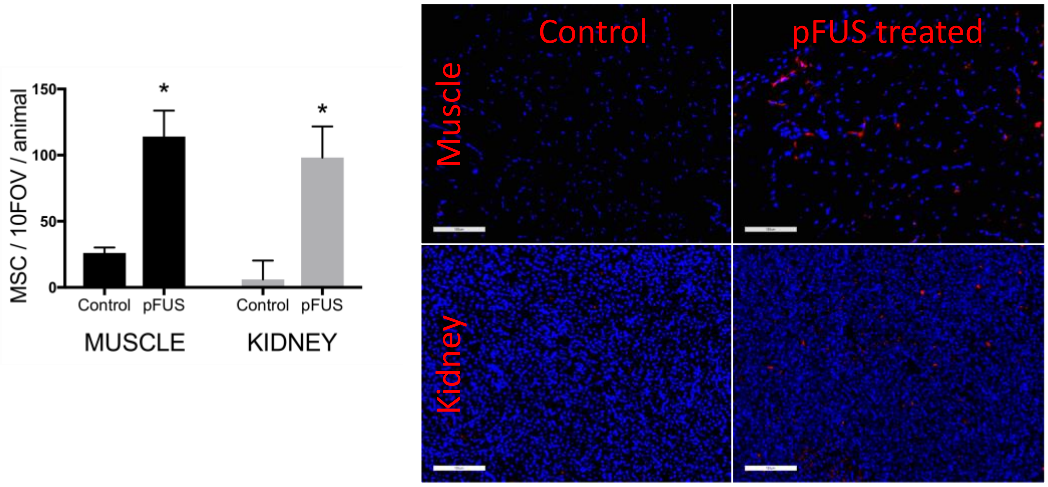

Supplemental Figure 3. pFUS induces MSC homing to sonicated muscle and kidneys in sv129 mice. This strain is the background wild-type that the TRPC1 knockout is derived from. pFUS, MSC infusions and detection were performed according to the Materials and Methods.

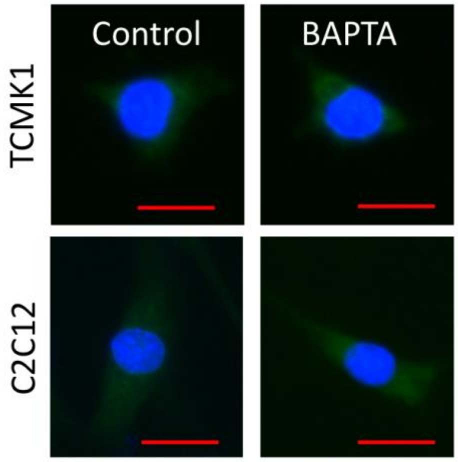

Supplemental Figure 4. BAPTA-AM loading into TCMK1 or C2C12 cells without pFUS treatment does not alter COX2 expression 4 hours after loading. COX2 shown in green and DAPI-stained nuclei shown in blue. Scale bars represent 10 μm.

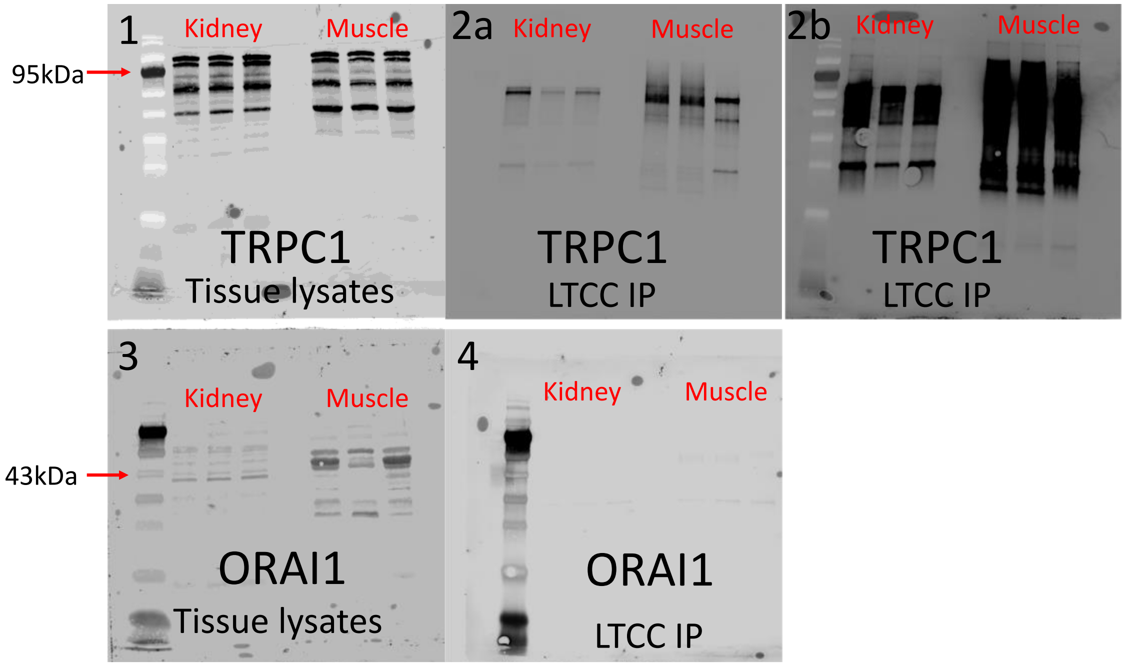

Supplemental Figure 5. Whole blot images for TRPC1 and ORAI1 before and after immunoprecipitation from kidney and muscle using the L-type Ca^{2+} channel antibodies. Blots 1 and 2 show TRPC1 whole tissue input (1) and subsequent pulldown with LTCC antibodies (2a). Blot 2b is an overexposure of 2a to reveal the molecular weight ladder. Blots 3 and 4 show ORAI1 from each tissue lysate (3) and subsequent pulldown with LTCC antibodies (4).

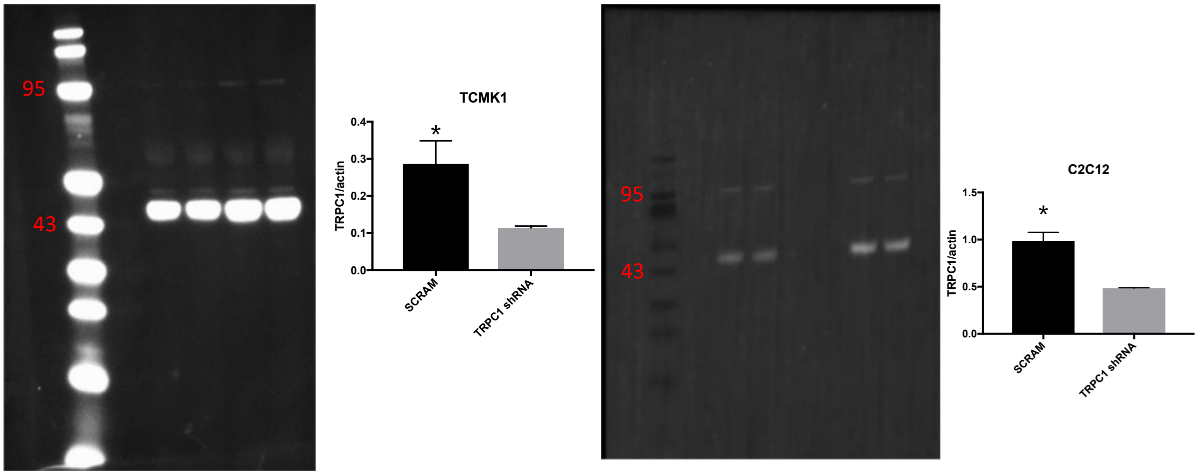

Supplemental Figure 6. Whole blot images for TRPC1 in TCMK1 cells (left) and C2C12 cell (right) following transfection with plasmids encoding shRNA against TRPC1 or control scramble shRNA sequences (SCRAM). Graphs show quantification of TRPC1 (95 kD) ratioed to b-actin expression (45 kD) and demonstrate that shRNA transfection reduces total TRPC1 expression in both TCMK1 and C2C12 cells.
